# The benefits of endurance exercise and Tai Chi Chuan for the task-switching aspect of executive function in older adults: an ERP study

**DOI:** 10.3389/fnagi.2014.00295

**Published:** 2014-10-28

**Authors:** Dong-Yang Fong, Li-Kang Chi, Fuzhong Li, Yu-Kai Chang

**Affiliations:** ^1^Physical Education Office, National Taipei University of TechnologyTaipei, Taiwan; ^2^Department of Physical Education, National Taiwan Normal UniversityTaipei, Taiwan; ^3^Oregon Research InstituteEugene, OR, USA; ^4^Graduate Institute of Athletics and Coaching Science, National Taiwan Sport UniversityTaoyuan, Taiwan

**Keywords:** cognitive function, executive control, fitness, physical activity, Taiji

## Abstract

This study was designed to determine the relationship between physical activity and the task-switching aspect of executive function by investigating the modulating roles of age, modality of physical activity, and type of cognitive function using behavioral and event-related potential (ERP) assessments. Sixty-four participants were assigned to one of four groups based on age and history of physical activity: older adults performing endurance exercise (OEE), older adults practicing Tai Chi Chuan (OTC), older adults with a sedentary lifestyle (OSL), and young adults (YA). Study participants completed a task-switching task under homogeneous and heterogeneous conditions while ERPs were recorded. The results revealed that YA had shortest reaction times compared with the three older adults groups, with OSL exhibiting the longest reaction time. YA also exhibited shorter P3 latency than OSL. No differences were observed in P3 amplitude between YA, OEE, and OTC; however, all three groups had significantly larger P3 amplitude compared with OSL in both task conditions. In conclusion, age and participation in physical activity influence the relationship between physical activity and task-switching, and a positive relationship was observed regardless of the modality of physical activity and type of cognitive function. Our ERP findings support the model of the scaffolding theory of aging and cognition (STAC) and suggest that regular participation in endurance exercise and Tai Chi Chuan may have equivalent beneficial effects on cognition at the behavioral and neuroelectric levels.

## INTRODUCTION

The U.S. consisted of 40.4 million adults older than 65 in 2010, representing 13% of the total population. The aged population in the U.S. is expected to double by 2040 and represent 20% of the total population. In other developed countries, the percentage of the older population is even greater than that in the U.S. The percentages of aged adults in Japan, Germany, Italy, France, U.K., and Russia ranged from 13.3 to 22.6% in 2010, and these numbers will increase to 22.8 to 34.4% by 2040. This change indicates a rapidly increasing number of older adults’ worldwide (Jacobsen et al., 2011). Aged adults generally exhibit decreases in physiological, biological, and behavioral functions, and these age-related declines extend to cognitive functioning. For example, negative linear trends in cognitive functions, such as speed of information processing, reasoning, and memory, and cognition-associated brain atrophy in the cerebellar hemisphere, lateral prefrontal cortex, and hippocampus have been observed in individuals between the ages of 20 and 80 years (Park and Reuter-Lorenz, 2009). The consequences of this age-related cognitive deterioration are related to the loss of the ability to live independently, impairment of day-to-day activities, deterioration of occupational and academic performance, and diminished health (Salthouse, 2010).

The cognitive decline that is impacted by aging depends on subdomains of cognition (Hoogendam et al., 2014); the executive function aspect of cognition is particularly susceptible to age-related deficits (Colcombe et al., 2005; Prakash et al., 2009). Executive function, also known as executive control, refers to a higher and more complex level of cognitive control processing that guides appropriate, purposeful, and goal-directed behaviors (Jurado and Rosselli, 2007). Executive function involves the inhibition of automatic and prepotent responses, which is necessary to maximize the level of performance required by the environment (Banich, 2009). Notably, executive function is an umbrella term and is believed to consist of several distinct subconstructs, such as inhibition, updating working memory, and task-switching (Miyake et al., 2000). Impairment of these executive functions has been recognized as a risk factor not only for neurodegenerative disorders, such as Alzheimer’s disease (Grober et al., 2008), but also for falling among older adults (Herman et al., 2010).

Positive relationships between physical activity and multiple cognitive functions have been well established. Long-term engagement in physical activity, including walking, is positively correlated with several cognitive functions (e.g., cognitive status, fluency, memory, and attention; Weuve et al., 2004). These beneficial impacts of physical activity on cognition are evident, even after considering the effects of possible confounders (e.g., age, depression, smoking, and heart disease; Etgen et al., 2010). Interestingly, exercise may disproportionately affect certain subdomains of cognitive function. A meta-analysis conducted by Colcombe and Kramer (2003) indicated that long-term exercise improved all cognitive functions in the speed, spatial, control, and executive function subdomains and that exercise exhibits the strongest effect on executive function in older adults. These findings led to the formulation of the selective improvement hypothesis (i.e., more beneficial for executive function), which has been supported by several studies (Hillman et al., 2004; Smiley-Oyen et al., 2008). However, the hypothesis was not supported by later meta-analytic reviews, in which exercise intervention led to similar modest improvements in executive function, processing speed, and memory (Smith et al., 2010) or larger effects on motor function and auditory attention but not executive function (Angevaren et al., 2008). These conflicting results may result from the specific aspect of executive function examined. The relationship between physical activity and different cognitive functions deserves further investigation.

Studies on physical activity and cognition have used event-related potentials (ERPs). An ERP is a time-locked electroencephalographic (EEG) measure that results from specific events and provides a non-invasive approach for detecting neuroelectric activity at high temporal resolution. ERPs also provide insight into the neural networks and cognitive operations that underlie explicit behavior, in which ERPs may offer a measure that is more sensitive than behavioral responses. Hillman et al. (2004) observed shorter reaction times during a flanker task in younger adults compared with older adults with histories of high physical activity, moderate physical activity, and being sedentary. This result suggests an age-related decline in the inhibition aspect of executive function. Although no significant differences were observed between reaction time and the level of physical activity among the older adults, those with high and moderate levels of physical activity demonstrated larger amplitudes than younger adults in the ERP P3 component in the inhibitory condition of the flanker task and shorter P3 latencies in the same task compared with older adults in the sedentary group. These findings suggest that the beneficial effect of participation in physical activity on inhibition can be demonstrated at a neuroelectric level, which in this case, included increased attentional resources and the acceleration of the speed of processing. A similar pattern of a physical activity-associated P3 has also been reported for the working memory aspect of executive function in older adults (Chang et al., 2013a).

Regarding inhibition and working memory, task-switching is recognized as another primary construct of executive function (Miyake et al., 2000). Task-switching, which is correlated with cognitive flexibility, is the ability to switch attention between different task settings (Kray et al., 2013). Tasks related to task-switching typically consist of homogeneous and heterogeneous blocks. Homogeneous blocks contain trials with a single rule set (e.g., AAAAA or BBBBB), and heterogeneous blocks contain trials with two or more rule sets (e.g., AABBAABB). Unlike homogeneous blocks that repeat the same rule, heterogeneous blocks require study participants to switch quickly between different task rules which involves more mental effort and results in longer reaction times. The difference in reaction times between heterogeneous and homogeneous blocks is termed the “global switch cost/mixing cost” (Monsell, 2003). This cost is believed to reflect the ability to maintain the instructions of two task settings and to adapt appropriate instructions for meeting the goal of the new task rule, both of which require more working memory (Kray and Lindenberger, 2000). It is important to investigate task-switching because it is a more predominant sub-construct of executive function than inhibition in older adults (Hull et al., 2008) and has been linked to physical activity (Hillman et al., 2006; Themanson et al., 2006; Kamijo and Takeda, 2010).

Some ERP studies involving relationships with physical activity have emphasized task-switching in older adults. Although physical activity is generally associated with behavioral and neuroelectric indices of task-switching performance, some equivocal results have been reported. Regarding age effects, Hillman et al. (2006) compared active and sedentary older adults with active and sedentary younger adults. They observed that the active groups of both age categories had smaller global switch costs, greater P3 amplitudes, and shorter P3 latencies compared with the sedentary groups. These findings suggest that long-term physical activity benefits task-switching performance, regardless of age. In contrast, physically active adults of both age categories had longer reaction times and slower post-error responses than the sedentary adults of both age groups; however, physically active older adults showed a smaller global switch cost and less neuroelectric activity than sedentary older adults. These differences were not observed in the younger adults (Themanson et al., 2006). These results suggest that both age and physical activity moderate the relationships between physical activity and executive function. Only marginally significant differences were observed in behavioral measures of performance on heterogeneous and homogeneous blocks, but significantly decreased P3 latencies were observed in the active groups compared with the sedentary groups, although only in heterogeneous blocks. This finding supports the selective improvement hypothesis at the neuroelectric level (Hillman et al., 2006). Conversely, other studies have indicated that the influence of physical activity either indirectly supports the selective improvement hypothesis (Themanson et al., 2006) or demonstrates general improvement in performance on both heterogeneous and homogeneous blocks (Dai et al., 2013). These inconsistent findings may result from different methodological designs. For example, Themanson et al. (2006) did not examine the condition effect (i.e., heterogeneous compared with homogeneous) and Dai et al. (2013) did not include a younger control group. Therefore, our study was designed to clarify the relationship between physical activity and task-switching performance by simultaneously investigating the influence of age and the types of cognition.

The modality of physical activity may also affect this relationship. Previous studies of the effects of exercise on cognition have focused predominantly on endurance exercise (e.g., walking and jogging), and a few recent studies have emphasized resistance exercise (Liu-Ambrose and Donaldson, 2009; Voss et al., 2011). Because recent animal studies have indicated that environmental enrichment leads to enhanced brain and cognitive plasticity (Pang and Hannan, 2013), exercise that involves multiple characteristics (e.g., exercise and cognitive demand) may be expected to benefit cognition. Using a task-switching paradigm, Dai et al. (2013), recently showed that older adults practicing closed-skill exercise (i.e., jogging and swimming) and open-skill exercise (i.e., table tennis and tennis) perform better under both homogeneous and heterogeneous conditions compared with their counterparts. The older adults in the open-skill group demonstrated additional benefits in global switch cost compared with the other two groups, suggesting that exercise that involves more physical and cognitive demands has greater benefits on specific cognition. The modality of physical activity should be given greater attention in future studies (Erickson and Kramer, 2009; Voss et al., 2011).

The present study focused on an alternative modality of physical activity, Tai Chi Chuan. Tai Chi Chuan, which is derived from traditional Chinese martial arts, is a multimodal mind-body exercise regimen (Chang et al., 2010) that benefits fitness, muscle strength, flexibility, postural control, and fall-risk reduction (Jahnke et al., 2010), as well as quality of life and well-being (Wang et al., 2010). The link between Tai Chi Chuan and cognition has recently been given more attention (Chang et al., 2010). Working from a neuroimaging perspective, Chang et al. (2014) proposed a model of a mechanism that suggests that the characteristics of Tai Chi Chuan (i.e., cardiovascular fitness, motor fitness, movement coordination, social interaction, and meditation) would benefit brain structure and function, which would, in turn, facilitate cognitive function. Links between cognition and additional factors of Tai Chi Chuan, such as training of sustained attentional focus, multitasking, and learning and memorization of new skills and movement patterns, have also been shown (Wayne et al., 2014). Recently, a beneficial effect of Tai Chi Chuan on cognition has been suggested from several narrative reviews (Chang et al., 2010, 2014; Miller and Taylor-Piliae, 2014). Furthermore, a randomized controlled trial conducted by Taylor-Piliae et al. (2010) indicated that Tai Chi Chuan not only benefits cognition but also improves cognition more than Western exercise. This greater effect was also revealed when comparing with other type of active controls (e.g., Western exercise, a combination of resistance and resistance exercise, or an educational program; Taylor-Piliae et al., 2010; Wayne et al., 2014). However, this line of research is nascent (e.g., proposing a temperate model, using pre-experimental designs, applying cognitive tasks with less precision), and no previous study has examined Tai Chi Chuan and cognition as assessed by task switching and measuring neuroelectric activity.

Accordingly, the present study had three purposes. The first aim was to examine the modulating roles of age and participation in physical activity on the relationship between physical activity and cognitive function, as measured at the behavioral and neuroelectrical levels. We hypothesized that although older adults would exhibit poorer performance at both levels of measurement compared with younger controls, the physically active older adults would demonstrate better cognitive performance than older adults with sedentary lifestyles. Second, we investigated the relationship between physical activity and different types of cognition, i.e., whether physical activity is associated with the “selective improvement hypothesis.” We assessed this possibility using the task-switching paradigm and predicted that physical activity would be strongly related to improved performance on tasks that required more cognitive control. Finally, we explored differences in performance and associated neuroelectric ERP processing on a task-switching task between older adults engaged in endurance exercise (e.g., walking) and Tai Chi Chuan to determine whether the modality of physical activity influences the relationship between physical activity and cognition. We predicted that older adults in both exercise modalities would exhibit more benefits to cognitive performance than older sedentary adults.

## MATERIALS AND METHODS

### PARTICIPANTS

Older adults (65–75 years of age) and younger adults (20–30 years of age) were enrolled using an advertisement that was posted in communities, universities, parks, jogging clubs, and Tai Chi Chuan clubs around Taipei, Taiwan. Participants in the experiments were required to meet initial criteria of right-hand dominance, normal or corrected-to-normal visual acuity, normal color vision, intact cognition [i.e., Mini-Mental State Examination (MMSE) > 26], and absence of current smoking, psychiatric disorders, neurological history, and head injury. The initial screening was assessed by a demographic questionnaire, a health screening questionnaire, and the MMSE.

In total, 48 older adults and sixteen younger adults who met the inclusion criteria were recruited. The eligible participants were placed into one of four groups based on their age and exercise status: OEE, OTC, OSL, and YA. The OEE and OTC participants were older adults who currently engaged in regular exercise (at least 5 years of exercise, three times a week and for 30 min each session). The exercise could be endurance exercise (i.e., walking and jogging) or the Yang style of Tai Chi Chuan. OSL participants were older adults who exercised irregularly (less than one 15-min session per week). The YA participants were younger adults who met the initial criteria. Additional screening was conducted with the International Physical Activity Questionnaire (IPAQ; Bauman et al., 2009) to confirm whether the recruiting process was appropriate. The IPAQ is a multi-country questionnaire designed to measure the amount of physical activity during the previous week, in which the mode, duration (i.e., at least 10 min), frequency, and intensity (i.e., light, moderate, or vigorous) of the exercise were reported. The metabolic equivalents (METs) of the reported exercise were calculated. All participants completed a written informed consent form. **Table 1** presents demographic data across four groups. The sample size met the requirement for power analysis in a 4 × 2 mixed design with power (0.80), alpha (0.05), and effect size f (0.48; Colcombe and Kramer, 2003). The research protocol was approved by the Institutional Review Board of the National Taiwan Sport University.

**Table 1:** Descriptive data of participants’ demographic and exercise characteristics (means ± SD).

Measures	Group			
	YA	OEE	OTC	OSL
Sample size	16	16	16	16
Gender (F/M)	6/10	10/6	8/8	8/8
Age (yrs)	22.43 ± 2.58^a^	68.37 ± 3.68	67.31 ± 4.92	68.93 ± 4.28
Education (yrs)	15.75 ± 1.43^a^	10.25 ± 3.10	11.68 ± 3.94	11.31 ± 3.92
Height (cm)	167.37 ± 6.91^a^	159.87 ± 8.09	159.75 ± 7.40	156.56 ± 5.95
Weight (kg)	60.06 ± 11.82	55.93 ± 8.11	59.78.8.82	56.12 ± 7.24
BMI (kg.m^-2^)	21.59 ± 3.56	21.80 ± 1.88	23.33 ± 2.37	22.85 ± 2.23
MMSE	29.87 ± 0.34^a^	28.37 ± 1.08	27.68 ± 1.25	28.25 ± 0.25
Exercise years	6.50 ± 4.69^b^	5.62 ± 5.48^b^	13.56 ± 8.61^a^	1.03 ± 2.03
Frequency/week	3.31 ± 1.19^b^	4.00 ± 1.03^b^	6.06 ± 1.23^a^	1.01 ± 1.41
Duration (session/min)	86.25 ± 24.18^a^	60.00 ± 17.32^b^	78.75 ± 15.00^a^	15.00 ± 21.21
**IPAQ**
Light	67.03 ± 179.58	1411.31 ± 703.94^a^	597.09 ± 151.28^b^	710.31 ± 313.20^b^
Moderate	1380.00 ± 1117.13^a^	397.81 ± 403.03	1910.00 ± 804.85^a^	60.00 ± 210.14
Vigorous	600.00 ± 919.13^a^	0.00 ± 0.00	0.00 ± 0.00	0.00 ± 0.00
Total	2047.03 ± 1743.21	1820.38 ± 524.81	2507.09 ± 1490.31	770.31 ± 397.40^a^

*YA, young adult; OEE, older endurance exercise; OTC, older Tai Chi Chuan; OSL, older sedentary lifestyle; BMI, body mass index; MMSE, Mini-Mental State Exam; IPAQ, International Physical Activity Questionnaire (metabolic equivalents/week); duration, duration of specific exercise (i.e., endurance exercise or Tai Chi Chuan).*

*^a,b^Represent the significant difference, *p* < 0.01.*

### TASK-SWITCHING TASK

Participants completed a modified task-switching task from Hillman et al. (2006). The task-switching task employed an S1–S2 paradigm in which an S1 (cue stimulus) appeared for 500 ms, followed by an S2 (target stimulus). The target duration was 1000 ms, and the inter-stimulus interval (ISI) was 1500 ms. The task-switching task consisted of two block types: homogeneous and heterogeneous.

The S1 of the first type of homogeneous block was a yellow triangle, and the S2 that followed was a numerical digit (i.e., 1, 2, 3, 4, 6, 7, 8, 9). Each participant in the block was instructed to identify whether the numeric stimulus was greater than or less than 5 by thumb pressing on the right or left button on a response box that was held in both hands. In the other type of homogeneous block, the S1 was a blue circle, and the participant was instructed to identify whether the numeric digit (S2) was odd or even by thumb pressing on the right or left button of the response box. Each block consisted of 90 target trials with identical percentages of correct right and left responses. In the heterogeneous block, the S1 was either a yellow triangle or a blue circle. If it was a yellow triangle, the participant was instructed to respond to the magnitude of the S2 by identifying whether the number was greater or less than 5. If the S1 was a blue circle, the participant was instructed to respond to the character of the S2 by identifying whether the number was odd or even. Each block consisted of 90 target trials with identical percentages of the two types of S1 and required right and left responses.

All stimuli were 4.5 cm × 4.5 cm and located in the center of a 37 cm × 37 cm computer monitor that was 100 cm from the participant. Each participant was administered several short practice trials for each block to familiarize the participant with the task prior to official testing (i.e., more than 90% of accuracy). Participants were required to complete five blocks of trials, two homogeneous blocks, and three heterogeneous blocks. Reaction time and accuracy on the homogeneous and heterogeneous blocks and the switch cost (i.e., subtracting the mean reaction time of the heterogeneous block from that of the homogeneous blocks) were calculated for the behavioral analysis.

### ERP RECORDINGS AND DATA REDUCTIONS

Ongoing EEG activity was continuously recorded using Ag/AgCl electrodes located at 14 scalp sites (i.e., Fz, F3, F4, Cz, C3, C4, Pz, P3, P4, T3, T4, Oz, O1, and O2) based on an international 10–20 system (Jasper, 1958). All electrodes were mounted using a Neuroscan Quick-Cap (Neuro, Inc. El Paso, TX, USA) and were referenced to right and left mastoid electrodes, with Fpz serving as the ground electrode. Vertical and horizontal electro-oculogram data (VEOG and HEOG) were collected from adhesive electrode sites below and above the left eye to monitor eye blinks and at sites on the outer canthus of each eye to monitor eye blinks and movements. All electrode impedances were kept below 5 kΩ. Neuroscan amplifiers (Neuroscan SynAmps II) were employed to amplify the EEGs, with a digitization rate of 1000 Hz, a band-pass filter of 0.1 to 50 Hz, and a notch filter of 60 Hz being applied to acquire data.

Event-related potentials were extracted by oﬄine processing that was segmented into 1200 ms epochs consisting of 200 ms prior to and 1000 ms after stimulus onset. Only corrected responses in the homogeneous and heterogeneous trials were averaged. The largest positive waveform between 300 and 550 ms was indexed as P3, and the P3 amplitude and latency at the midline of Fz, Cz, and Pz were identified and analyzed (Chang et al., 2013a; Dai et al., 2013). The topographic map of P3 resulted from 32 channels is presented.

### STATISTICAL ANALYSIS

One-way analysis of variance (ANOVA) or a Chi Square test was initially used to test the significance of group differences in demographic and exercise-related characteristics between the four groups where appropriate. Two-way repeated-measure ANOVAs with a mixed 4 (Group: YA, OEE, OTC, or OSL) × 2 (Condition: homogeneous or heterogeneous) model were separately employed for the behavioral data (i.e., reaction time and accuracy), and a one-way ANOVA was computed for the switch cost of behavioral data as expressed by reaction time and accuracy. Three-way repeated-measure ANOVAs with a 4 (Group) × 2 (Condition) × 3 (Site: Fz, Cz, or Pz) mixed model were then separately employed for the amplitude and latency of the ERP P3 component. All ANOVAs were subjected to a Greenhouse–Geisser adjustment to meet the assumption of sphericity. Main and interaction effects were followed by the Tukey’s HSD (honestly significant difference) *post hoc* test and multiple comparisons of *t*-tests with Bonferroni corrections when necessary. Effect size was expressed as a partial eta-squared (η^2^) to determine the magnitude of the effect when a significant main and interaction effects was reached. An alpha of 0.05 was used as the level of significance prior to the Bonferroni correction.

## RESULTS

### PARTICIPANTS

A one-way ANOVA revealed no significant differences between the four groups in terms of weight, body mass index (BMI; *ps* > 0.18), or gender (chi-squared > 0.10). However, significant differences between the four groups were observed in age, education, height, MMSE, exercise years, exercise frequency per week, and exercise duration per session [*Fs*(3,60) > 6.61, *ps* < 0.001). The *post hoc* test revealed that YA participants were younger and taller, had more years of education, and had a higher MMSE than the OEE, OTC, and OSL participants (*ps* < 0.004), whereas no significant differences in these variables were observed between the three older adult groups (*ps* > 0.10). OTC had the most exercise years and the highest frequency of exercise per week. YA and OEE ranked next, and OSL had the least exercise years and lowest frequency of exercise per week. YA and OTC had the longest durations per session. OEE ranked next, and OSL ranked fourth.

One-way ANOVAs also revealed significant differences between the four groups in light-intensity, moderate-intensity, and vigorous-intensity exercise and in the total IPAQ score [*Fs*(3,60) > 6.07, *ps* > 0.001]. The *post hoc* test revealed that OEE had the highest MET in the light-intensity IPAQ score; OSL and OTC ranked next, and YA ranked last. OTC and YA had the highest MET in the moderate-intensity IPAQ score, and OEE and OSL ranked next. YA had a higher MET in the vigorous-intensity IPAQ score than the three older adult groups. Lastly, OTC, YA, and OEE had higher METs in the overall IPAQ score than OSL.

### BEHAVIORAL DATA

#### Reaction time

A two-way ANOVA revealed a main effect of group [*F*(3,60) = 30.31, *p* < 0.007, η^2^ = 0.60]. YA had the shortest reaction time, followed by OEE and OTC, and OSL had longest reaction time. A main effect of condition was also observed [*F*(1,60) = 253.75, *p* < 0.0001, η^2^ = 0.81]. The homogeneous condition had shorter reaction times than the heterogeneous condition. An interaction of group and condition was observed [*F*(3,60) = 8.04, *p* < 0.0001, η^2^ = 0.28].

**FIGURE 1:**
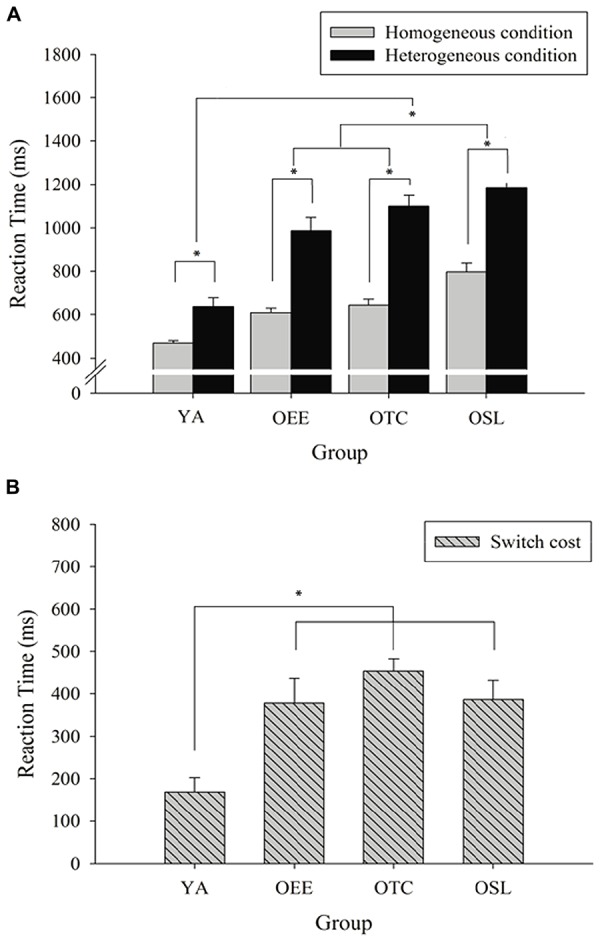
**Reaction times (mean ± SE) for young adults (YA), older adults practicing endurance exercise (OEE), older adults practicing Tai Chi Chuan (OTC), and older adults with a sedentary lifestyle (OSL). (A)** Homogeneous and heterogeneous conditions. **(B)** Switch cost. *p < 0.007.

Regarding the interaction of group and condition, the *post hoc* test for the homogeneous condition revealed that YA had the shortest reaction time (496.56 ± 42.09 ms, *ps* < 0.004). OEE and OTC had the second shortest reaction times, with no significant differences (608.62 ± 89.87 and 645.78 ± 107.30 ms, respectively), and OSL had the longest reaction time (796.82 ± 162.65 ms). A similar ranking of reaction time was detected for the heterogeneous condition. YA had the shortest reaction time (638.39 ± 163.33 ms, *ps* < .0001), OEE and OTC had the second shortest reaction times (986.76 ± 247.96 and 1099.75 ± 198.48 ms, respectively), with no significant differences between the groups, and OSL had the longest reaction time (1183.24 ± 169.45 ms). Significantly shorter reaction times for the homogeneous condition than for the heterogeneous condition were observed for the four groups (*p* < 0.001; **Figure 1A**).

Regarding switch cost, a one-way ANOVA revealed significant differences between the four groups [*F*(3,60) = 8.04, *p* < 0.0001]. The *post hoc* test revealed that YA had a smaller switch cost (168.84 ± 133.20 ms, *p* < 0.004) than OEE, OTC, and OSL (378.14 ± 236.78, 453.93 ± 144.55, and 386.42 ± 185.57 ms, respectively), with no significant differences between the three older adult groups (**Figure 1B**).

#### Accuracy

A two-way ANOVA revealed a main effect of group [*F*(3,60) = 4.46, *p* < 0.007, η^2^ = 0.18] where YA had higher accuracy than OSL (94.60 ± 0.02, 73.30 ± 0.02, *p* < 0.03). A main effect of condition was observed [*F*(1,60) = 46.89, *p* < 0.0001, η^2^ = 0.44] where the heterogeneous condition had less accuracy than the homogeneous condition. An interaction of group and condition was also observed [*F*(3,60) = 4.42, *p* < 0.007, η^2^ = 0.18].

The interaction of group and condition was examined with a *post hoc* test. No significant differences between the four groups were observed for the homogeneous condition (accuracy ranged from 93.88 ± 0.07 to 97.56 ± 0.05, *p* > 0.05). However, the *post hoc* test for the heterogeneous condition revealed that YA had higher accuracy than OSL (93.69 ± 0.06, 72.75 ± 0.22, *p* < 0.03). No other significant differences were observed in the heterogeneous condition. Additionally, OEE, OTC, and OSL (*ps* < 0.009) but not YA were significantly more accurate in the homogeneous condition than the heterogeneous condition.

Regarding switch cost, a one-way ANOVA revealed a significant difference between the four groups [*F*(3,60) = 4.45, *p* < 0.007]. A *post hoc* test revealed that YA had a lower switch cost (-0.02 ± 0.05, *p* < 0.05) than OEE, OTC, and OSL (-0.14 ± 0.19, -0.15 ± 0.11, and -0.21 ± 0.21, respectively, *p* < 0.01), but no significant differences were observed between the three older adult groups.

### ERP DATA

#### P3 Amplitude

**FIGURE 2:**
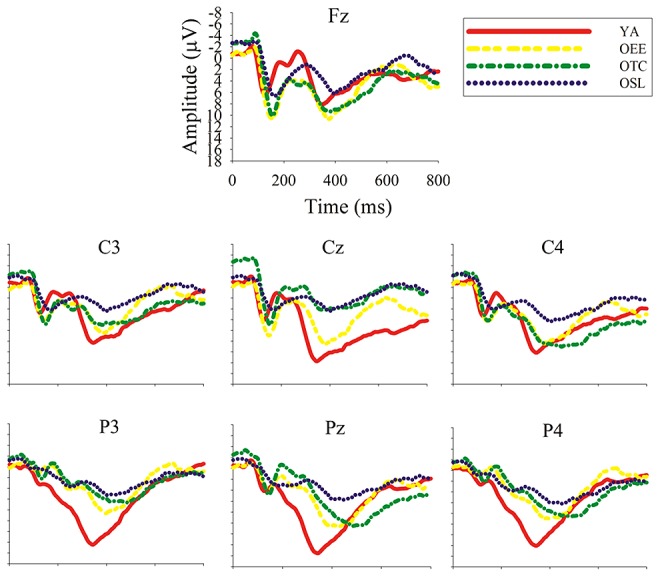
**Grand average ERP waveforms for the averaged homogeneous and heterogeneous conditions for the four groups at seven electrode sites**.

A three-way ANOVA revealed a main effect of group [*F*(3,60) = 6.09, *p* < 0.001, η^2^ = 0.23]. OSL had a smaller P3 amplitude (10.83 ± 1.32 μV) than OEE, OTC, and YA (17.08 ± 1.32, 18.02 ± 1.32, 16.71 ± 1.32 μV, respectively), and no significant differences were observed between the latter three groups (*p* > 1.00; **Figure 2**). Interactions of group and condition [*F*(3,60) = 3.03, *p* < 0.04, η^2^ = 0.13] and group and site [*F*(6,120) = 5.60, *p* < 0.0001, η^2^ = 0.22] were also observed.

The interaction of group and condition was examined with a *post hoc* test. For the homogeneous condition, OSL had a smaller P3 amplitude (10.53 ± 3.91 μV, *p* < 0.04) than OEE, OTC, and YA (19.27 ± 8.94, 18.59 ± 4.47, 16.32 ± 5.06 μV, respectively), and no significant differences were observed between the latter three groups. Similar results were observed for the heterogeneous condition, in which OSL had a smaller P3 amplitude than OEE, OTC, and YA (11.12 ± 5.18 μV vs. 17.44 ± 7.32 μV, and 17.09 ± 4.74 μV, respectively, *p* < 0.03). Lastly, OEE but not OSL, OTC, or YA had significantly larger P3 amplitude in the homogeneous condition than the heterogeneous condition (*p* < 0.003).

**FIGURE 3:**
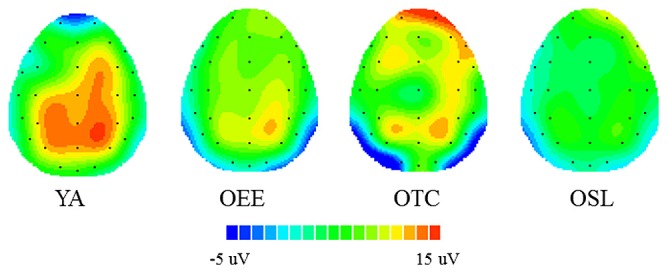
**Topographical maps of the mean P3 voltage (300–550 ms) for the averaged homogeneous and heterogeneous conditions for the four groups**.

The interaction of group and site was examined with a *post hoc* test. No significant differences were observed at Fz, but OSL had a smaller P3 amplitude at Cz (10.17 ± 4.04 μV, *p* < 0.02) than the other three groups (16.96 ± 6.84 μV, 18.83 ± 6.51 μV, and 18.42 ± 5.75 μV for OEE, OTC, and YA, respectively). OSL also had a smaller P3 amplitude at Pz (9.71 ± 3.04 μV, *p* < 0.001) than the other three groups (16.52 ± 5.88 μV, 17.77 ± 5.97 μV, 18.98 ± 4.86 μV for OEE, OTC, and YA, respectively). However, significant within-group site differences were observed only in YA, in which Pz > Cz > Fz (*p* < 0.001; **Figure 3** presents topographical maps of the mean P3 voltage).

#### P3 Latency

A three-way ANOVA revealed a main effect of group [*F*(3,60) = 3.59, *p* < 0.019, η^2^ = 0.19]. YA had a shorter P3 latency (387.00 ± 18.17 μV) than OSL (463.14 ± 18.17 μV), but no significant differences were observed between the three older groups. A main effect of site [*F*(2,120) = 4.55, *p* < 0.012, η^2^ = 0.07], an interaction of group and site [*F*(6,120) = 3.03, *p* < 0.0001, η^2^ = 0.23], and an interaction of condition and site [*F*(2,120) = 7.64, *p* < 0.0021, η^2^ = 0.11] were also observed.

The interaction of group and site was examined with a *post hoc* test. No significant differences were observed at Fz or Cz, but YA had the shortest P3 latency at Pz (350.77 ± 53.69 ms, *p* < 0.01), OEE and OTC had the second shortest at Pz (441.72 ± 44.57 ms and 420.79 ± 76.44 ms, respectively), and OSL had the longest at Pz (498.23 ± 87.24 ms, *p* < 0.01). Significant within-group site differences were observed in YA, which had a shorter latency at Pz than Fz, and OEE and OSL had longer latencies at Pz than Fz (*p* < 0.01). No significant differences were observed within the OTC group.

The interaction of condition and site was examined with the *post hoc* test. No significant site effects were observed for the homogeneous condition, but in the heterogeneous condition, Pz (435.31 ± 99.63 ms, *p* < 0.001) had a longer latency than Cz and Fz (396.70 ± 105.63 ms and 387.66 ± 108.87 ms, respectively). A significant difference between the homogeneous and heterogeneous conditions was identified at the Fz site (421.43 ± 106.17 ms compared with 387.06 ± 108.87 ms, *p* < 0.02), but no significant differences were observed at Cz or Pz.

## DISCUSSION

Previous studies have demonstrated a positive relationship between endurance exercise and cognition. The present study was designed to extend these studies by exploring the influences of age, participation in physical activity, type of experimental condition, and modality of physical activity in the relationship between physical activity and the task-switching aspect of executive function at behavioral and neuroelectric levels.

Our results revealed robust age effects; older adults exhibited poorer performance in the behavioral measures (e.g., reaction time, accuracy, and switch cost) than younger individuals. These findings corroborate previous empirical studies (Hillman et al., 2006) and a meta-analysis that indicated that age-related deterioration is evident in cognitive tasks that involve task-switching (Wasylyshyn et al., 2011). Although no differences were observed in an index of switch cost between the three older adult groups, OEE and OTC demonstrated superior performance (i.e., shorter reaction times in both the homogeneous and heterogeneous conditions) compared with OSL, indicating that participation in physical activity positively influences task switching. These findings are partially consistent with previous studies that have demonstrated that walking exercise enhances task-switching performance (Kramer et al., 1999) and that older adults who engaged in regular closed- or open-skill exercises have better task-switching performance than counterparts who do not exercise (Dai et al., 2013). The present findings are also similar to those of previous studies that showed that younger and older adults who exercised regularly performed better than sedentary controls (Hillman et al., 2006). Together, these results tentatively support our first hypothesis that age and participation in physical activity influence the relationship between physical activity and task-switching performance at behavioral levels; i.e., aging tends to impair cognitive performance, but physical activity tends to ameliorate age-related cognitive deterioration.

An age effect was also observed in the ERP results. YA had a larger P3 amplitude and a shorter P3 latency than the older adults, especially those in the OSL group. The P3 component has been associated with aging (Friedman et al., 1997; Walhovd and Fjell, 2003). In a cross-sectional comparison of adults of 20–79 years of age, Picton et al. (1984) found that P3 amplitude was reduced at a rate of 0.18 μV and that P3 latency was increased at a rate of 1.36 ms for every additional year of age. Our results also show an interaction between group and site. YA had the largest P3 amplitude at Pz, but OSL showed no differences at Pz, Cz, or Fz. These findings are consistent with the “compensation hypothesis,” which is an ERP-related aging hypothesis. The compensation hypothesis suggests that the brain cortex, particularly in the frontal scalp area, is overactivated during task performance in older adults compared with younger adults, which reflects the requirement of older adults to recruit more frontal activation of neural networks to compensate for age-related cognitive deterioration (Friedman et al., 1997; Friedman, 2003). The compensation demonstrated by the ERP data was a downstream form of the frontal compensation or the posterior–anterior shift in aging that has been proposed by functional neuroimaging studies (Davis et al., 2008; Reuter-Lorenz and Park, 2010).

Potentially the most intriguing finding of the present study is that OEE and OTC not only exhibited larger P3 amplitudes than OSL but that their P3 amplitudes were equivalent to that of YA. This result suggests that older adults who are engaged in endurance exercise and Tai Chi Chuan are similar to younger adults at the neuroelectric level. The positive association between physical activity and ERP indices on task-switching identified in the present study is similar to the results of previous studies that examined the effects of exercise on inhibition and working memory across the lifespan (Hillman et al., 2004; Chang et al., 2013a,b). Because P3 amplitude is associated with the mental representation of a deviant event (Donchin, 1981) and believed to reflect the amount of attentional resources allocated to a given task (Polich, 1987; Polich and Heine, 1996) and because P3 latency is believed to be associated with the duration of stimulus evaluation and classification (Donchin, 1981), the beneficial effect of physical activity on task-switching may be related to improved attentional resource allocation rather than the speed of stimulus evaluation and classification. Notably, our findings support the conceptual model of the STAC proposed by Park and Reuter-Lorenz (2009). According to STAC, aging-related cognitive function is primarily associated with neural challenges, functional deterioration, and compensatory scaffolding. Although aging has a negative effect on cognition and brings neural challenges (e.g., white matter changes and cortical thinning) and functional deterioration (decreased medial temporal recruitment), the compensatory scaffolding can be enhanced by functional compensatory mechanisms (e.g., frontal recruitment and neurogenesis) and environmental alterations (e.g., new learning and exercise). Therefore, our ERP results support the STAC model by showing that all of the older adult groups recruited more brain resources than the younger adults and that older adults who were engaged in physical activity exhibited P3 patterns similar to those of younger adults, suggesting that although aging negatively affects cognition, physical activity provides a scaffold for improving cognitive function. Taken together, these results again support our first hypothesis that age and participation in physical activity influence the relationship between physical activity and task-switching at both behavioral and neuroelectric levels. Specifically, although aging is associated with reduced cognitive performance, physical activity compensates to some degree for the age-related cognitive deterioration observed in task-switching, particularly as expressed at the neuroelectric level.

This study also attempted to investigate the selective improvement hypothesis, which postulates a positive association between physical activity and cognitive tasks, particularly those that involve executive function. A significant main effect of condition was observed in terms of longer reaction times and less accuracy in the heterogeneous condition than the homogeneous condition, replicating previous studies in which the heterogeneous task required a greater amount of executive control. Although an interaction of group and condition was observed, follow-up analysis revealed that the four groups had similar performance order, regardless of task conditions; i.e., the YA performed best, followed by OEE and OTC, and OSL performed worst in both homogeneous and heterogeneous conditions. Although ERPs are not directly comparable to behavioral indices, the P3 amplitude exhibited a similar pattern. Specifically, YA, OEE, and OTC showed larger P3 amplitudes than OSL for both task conditions. These results suggest that physical activity tends to be associated with general rather than selective cognitive improvement. Although the results differ somewhat from our hypothesis and previous studies (Kramer et al., 1999; Colcombe and Kramer, 2003; Hillman et al., 2006), improved general cognitive function was also observed in several studies that measured both general and selective improvement (Colcombe and Kramer, 2003) or general improvement only (Smith et al., 2010; Dai et al., 2013). Animal studies have indicated that physical activity leads to improvement in cognition-related molecular constructs, such as neurogenesis (Uda et al., 2006) and synaptogenesis (Eadie et al., 2005; Kempermann, 2008). Physical activity has also been linked to increased neurotrophic factors, such as brain-derived neurotrophic factor (BDNF; Cotman and Engesser-Cesar, 2002), and relationships between exercise, BDNF, and cognition have recently been established in human studies (Kim et al., 2011; Coelho et al., 2013). Because these molecular constructs are the foundation of a variety of cognitive functions, increased molecular alterations may be the underlying mechanism for improved general cognitive function related to physical activity.

Our third goal was to determine whether the physical activity modality influences the relationship between physical activity and cognitive function. We found that physical activity in general, not the specific modality of physical activity, improved task-switching performance, which suggests that physical activity positively influences cognition, regardless of the type of physical activity. Although these results should be interpreted with caution due to the small sample size, they are consistent with previous studies that independently examined endurance exercise (Angevaren et al., 2008; Smith et al., 2010) and Tai Chi Chuan (Chang et al., 2010, 2014; Miller and Taylor-Piliae, 2014; Wei et al., 2014) on cognition and brain. The present study extends this knowledge by comparing two exercise modalities. Although both types of physical activity were positively associated with cognitive function at the behavioral and neuroelectric levels, their underlying mechanisms may differ. Kramer et al. (1999) indicated that six months of walking exercise training not only enhanced cardiovascular fitness (+5.1%) but also improved task-switching performance in older adults, suggesting that cardiovascular fitness may be the link between exercise and cognition. Later studies using magnetic resonance imaging have further suggested that older adults who were more fit or who participated in long-term endurance exercise training had larger gray and white matter areas (which are ordinarily vulnerable to aging) than counterparts with lower fitness levels (Colcombe et al., 2006; Gordon et al., 2008) and that these individuals had more cortical activation in the brain regions involved in attentional control (e.g., frontal and parietal cortices; Colcombe et al., 2004) than matched control groups. These findings suggest the importance of cardiovascular fitness in the relationship between exercise and cognition. However, although Tai Chi Chuan has also been associated with better cardiovascular fitness (Taylor-Piliae and Froelicher, 2004), this type of exercise has also been linked to improvements in other fitness variables, such as muscle strength and endurance, balance, flexibility, and coordination (Leung et al., 2011; Lu et al., 2013) because it involves slow movement, endurance, a low stance, and coordination. These factors may have different effects on cognitive processes and the brain. For example, older adults with both higher cardiovascular fitness and higher motor fitness exhibit better executive control performance compared with counterparts with lower fitness status. The higher cardiovascular fitness group showed enhanced activity in the brain regions primarily involved in executive control, whereas the higher motor fitness group showed enhanced activity in the brain regions primarily involved in visuospatial processes (Voelcker-Rehage et al., 2010). Tai Chi Chuan training also involves opportunities to interact with individuals within a group, and social interaction may also influence the brain and cognition. Mortimer et al. (2012) reported increased executive control performance and whole-brain volume following membership in a Tai Chi Chuan group and a social interaction intervention group, whereas these changes were not observed in either a walking group or a non-exercise group. Members of the Tai Chi Chuan group had larger changes than members of the social interaction group. Although the lack of changes in the walking group may have resulted from the walkers’ lower quantity of exercise, these differences suggest a specific influence of Tai Chi Chuan. In summary, our findings indicate that both endurance exercise and Tai Chi Chuan improve cognitive function. The underlying mechanisms may differ because different specific exercise characteristics are involved. These results warrant further investigation.

### LIMITATIONS AND FUTURE DIRECTIONS

The present study has some limitations that should be considered when interpreting the results. Because of the cross-sectional nature of the design, a direct causal link between exercise and improved cognitive function cannot be confirmed. Although a longitudinal study comparing the effect of exercise modality on cognition has not yet been published, some studies have examined influences on cognitive or neurocognitive function after long-term programs of aerobic exercise (Colcombe et al., 2006), resistance exercise (Liu-Ambrose et al., 2010), and Tai Chi Chuan (Li et al., 2013). These studies provide the foundations and suggest possibilities for discovering a causal relationship between the modality of exercise and cognitive function. Such studies should be conducted in the future. Additionally, although the effects of exercise-related variables (e.g., exercise years, frequency per week, duration, and overall amount of physical activity) were consistent with our hypotheses, which suggested that our group assignments were appropriate, fitness is only inferred, and we cannot specify which fitness-related variables (e.g., cardiovascular and motor fitness) affected cognition. Different modalities of exercise may enhance specific types of fitness rather than overall general fitness (e.g., aerobic exercise may primarily improve cardiovascular fitness and Tai Chi Chuan may primarily improve motor fitness), and as noted above, these specific types of fitness have been linked to different cognitive and brain functions (Voelcker-Rehage et al., 2010). Future research that assesses these fitness-related variables may provide a better understanding of the relationships between modalities of exercise and cognition. Third, the thumb pressing response task requires participants to generate sufficient mechanical coupling from the thumb muscles. A study using electromyography revealed that the response time for motor maneuvers varies between older adults, which may constitute a confounding variable in determining the reaction time of the cognitive task (Tsang and Hui-Chan, 2003). Therefore, the possibility of a motor element associated with aging should be considered when applying this type of cognitive task in future studies examining older populations. Another potential limitation of the present study is that we primarily recruited healthy older adults with intact cognition, and the finding of a positive association between exercise and cognition may not apply to other populations. However, previous large-scale prospective studies have suggested inverse associations between physical activity and the risk of cognitive decline (Sofi et al., 2011) and between physical activity and the risk of neurodegenerative diseases, such as dementia, Alzheimer’s disease, and Parkinson’s disease (Hamer and Chida, 2009). Meta-analytic reviews have shown that exercise has a larger overall effect on cognition in older adults with cognitive impairment and dementia (ES = 0.57, Heyn et al., 2004) than in older adults with healthy cognition (ESs = 0.12–0.48, Colcombe and Kramer, 2003; Smith et al., 2010). Although these meta-analyses primarily investigated the effects of aerobic exercise, these positive findings suggest the possibility of exercise modality effects in older adults who exhibit a wide range of cognitive function. Lastly, future studies are encouraged to consider characteristics of exercise participation, such as Tai Chi Chuan/jogging in a group or individually, to examine whether Tai Chi Chuan participants perform self-directed practice or instructor-directed practice with/without verbal cues (Wayne and Kaptchuk, 2008), or to recruit Tai Chi Chuan practitioners with more extensive training or longer training years (e.g., more than 10 years) which may develop better quantified criterion to evaluate the degree of Tai Chi Chuan exercise. The different characteristics and training approaches may influence cognition, particularly the task-switching aspect of executive function.

## CONCLUSION

To the best of our knowledge, the present study is the first to explore the association between physical activity and task-switching by examining the modulating roles of age, physical activity modality, and type of cognition using both behavioral and neuroelectric approaches simultaneously. In conclusion, the present ERP findings suggest that age and physical activity influence the relationship between physical activity and task-switching. The positive effect of physical exercise on cognition may be independent of the modality of physical activity and the type of executive control processes. Although age-related cognitive decline is to be expected in later years, older adults who engage in physical activity (either endurance exercise or Tai Chi Chuan) exhibit benefits in many different neurocognitive domains.

## Conflict of Interest Statement

The authors declare that the research was conducted in the absence of any commercial or financial relationships that could be construed as a potential conflict of interest.
